# How do people who use drugs receiving Opioid Medication Therapy perceive their treatment ? A multicentre study

**DOI:** 10.1186/s12954-022-00608-6

**Published:** 2022-03-28

**Authors:** Morgane Guillou Landreat, Antoine Dany, Gaelle Challet Bouju, Edouard-Jules Laforgue, J. Cholet, Juliette Leboucher, Jean Benoit Hardouin, the OPAL Group, Pierre Bodenez, Marie Grall-Bronnec, Morgane Guillou-Landreat, Bertrand Le Geay, Isabelle Martineau, Philippe Levassor, Paul Bolo, Jean-Yves Guillet, Xavier Guillery, Corine Dano, Caroline Victorri Vigneau, Marie Grall Bronnec

**Affiliations:** 1 INSERM UMR 1246, SPHERE, Methods in Patient- Centered Outcomes and Health Research, Nantes and Tours Universities, Nantes, France; 2 EA 7479 SPURBO, BREST University, Brest, France; 3 CHU Nantes, Addictology and Psychiatry Department, Nantes, France; 4 CHU Nantes, Pharmacology Department, Nantes, France; 5 Hôpital Saint Jacques, 44000 Nantes, France

## Abstract

**Background:**

The resurgence of heroin use and the misuse of pharmaceutical opioids are some of the reasons for a worldwide increase in opioid dependence. Opioid Medication Therapies (OMT) have amply demonstrated their efficacy. From a medical point of view, the main objectives of OMT concern medical and social outcomes, centred on risk reduction and the cessation of opioid use. But patient points of view can differ and few studies have explored opioid-dependent patient viewpoints on their OMT. This variable seems important to consider in a patient-centred approach. The aim of our study was to explore points of view of people who use drugs (PWUD) treated with OMT, in a large multicentre sample.

**Method:**

A cross-sectional multicentre study explored the points of view of PWUD with Opioid Use Disorder following OMT. Data regarding the patients’ points of view were collected using a self-administered questionnaire developed by the scientific committee of the study. A descriptive analysis and an exploratory factor analysis were performed to explore the structure of items exploring patient viewpoints.

**Results:**

263 opioid dependent PWUD were included, a majority were men consuming heroin prior to being prescribed OMT. 68% were on methadone, 32% were on buprenorphine. Most PWUD identified a positive impact on their lives, with 92.8% agreeing or strongly agreeing that OMT had changed a lot of things in their lives. The exploratory factor analysis identified three factors: (F1) items related to points of views concerning the objectives and efficacy of OMT; (F2) items related to the legitimacy of OMT as a treatment compared to a drug, (F3) items related to experiences and relationships with OMT.

**Conclusion:**

Patient viewpoints on efficacy were correlated with the pharmacological benefits of OMT and with the associated psychosocial measures. The implications of OMT in relationships, such as the feeling of being judged, concerned a majority. Points of view were ambivalent concerning the role of OMT as a treatment or as a drug. Involving patient points of view in therapeutic strategies decisions could help enhance positive views among PWUD on OMT and help PWUD towards their recovery.

*Trial registration:* OPAL study was registered: (NCT01847729).

## Background

The resurgence of heroin use in some countries such as the United States, and the misuse of pharmaceutical opioids, such as fentanyl, are some of the reasons for the current “opioid crisis” [1–3]. The number of people with opioid dependence worldwide increased from 18.2 million in 1990 to 26.8 million in 2016 [4]. In Europe, the mean prevalence of opioid use disorders is estimated to be between 3.6 and 4.4 per thousand inhabitants (15–64 years of age). OUD are associated with increased early mortality and morbidity, due to a dramatic increase in lethal overdose rates [5], and are a growing public health concern. In addition, opioid-dependent PWUD present more comorbidities than the general population (e.g. abscesses, infective endocarditis, etc.) [6–19]. More particularly, infectious diseases, such as Hepatitis C. or HIV infection are a major area of concern in PWUD. HIV infection has been seen among persons who inject drugs in 61 countries [20]. Development of a “combined prevention” approach, significantly reduced new HIV infections among PWUD in several locations including New York City, Vancouver and France [20]. The efforts effectively ended the local HIV epidemic among persons who inject drugs in those locations, in France in 2010, the estimated prevalence of HIV in PWUD was 10% [21]. But HCV remains still disproportionately prevalent in PWUD. HCV prevalence in France was extremely high before the introduction of the combined prevention model (> 70%), it remains still over 40% in the last estimate [21]. The World Health Organization estimates the global burden of harm at 9.2 and 11.2 million disability-adjusted life-years for opioid use and opioid dependence, respectively [22, 23].

OUD is associated with rapidly-appearing, strong withdrawal symptoms and craving, both responsible for continuing opioid use and unsuccessful attempts to stop consumption, despite PWUD s' awareness of the damage [24]. OUD treatment includes psychosocial treatment combined with medication [25]. In France, two medications are available for opioid maintenance therapy (OMT): buprenorphine (BHD) and methadone (MTD): BHD is a partial mu agonist that can be prescribed by any physician and MTD is a full mu agonist, listed as a narcotic, its initiation is restricted to physicians from specialized units or hospitals. The use of these medications is superior to other treatment options for most PWUD with OUD, for maintenance treatment, and MTD and BHD are equally efficacious [26, 27]. In France, 170,000 PWUD are currently being treated for opioid dependence, of whom 65% are on BHD, and 35% on methadone [28]. These OMTs have amply demonstrated their efficacy [29, 30].

Both OMT drugs have proved to be efficacious in terms of public health, access to care and risk reduction, particularly in France where buprenorphine is widely available on prescription by any physician [25, 30, 31]. The choice of the OMT prescribed depends on many variables. The first is access to treatment, depending on policies [25], and the implication and training of physicians [30, 32]. The second variable concerns the patients’ personal vulnerabilities and addictive history (patterns of heroin use, psychiatric comorbidities, failure of previous treatments etc.) [25, 33]. The third is the patients’ acceptance of OMT, which depends on their personal choices and points of view about the treatment [34]. The patients’ implication is a major variable in the success of OUD treatment [35, 36]. The choice among the available treatment options should be a shared decision between the clinician and the patient, in a patient-centred approach [25].

OMT prescription has different objectives: (1) to avoid opioid withdrawal symptoms, (2) to block the effects of illicit opioids, (3) to reduce opioid craving, to stop or reduce the use of illicit opioids and to prevent relapse, (4) to stop drug injection to reduce risks of infection, (5) to promote and facilitate patient commitment to recovery-oriented activities including psychosocial interventions [33, 37, 38]. Prescriber objectives are centred on these medical and social objectives, aiming to reduce risks and stop opiate use, with the exception of OMT. Leading causes of death among people with opioid dependence include unintentional drug overdose, suicide, and HIV and Hepatitis C infections [39]. Prescribers are aware of these severe risks associated with the persistent use of opioids. In addition, OMT is a long-term treatment, sometimes for life, and recommendations underline the need to maintain OMT over a long period of time [25, 33]. The long term use of OMT is a way of reducing the high risk of mortality due to opioid overdose [40], estimated at 30 deaths/1000 person-years in the 4 weeks after OMT cessation [41]. But patients’ objectives and perspectives may differ. A previous study underlined that PWUD focused on the side effects of OMT (sweating, libido disorders, weight gain, etc.) which can deteriorate their quality of life [42]. In addition, as previously underlined in the literature [43], PWUD with OMT expressed the feeling that being kept on maintenance treatment made them feel they were still "drug addicts", while in the medium and long term, PWUD mostly hoped to stop all opiates, including OMT [42, 44]. So there are two opposite points of view. A previous study compared these points of view and came to the conclusion that prescribers wanted their "PWUD to survive, whereas PWUD wanted to live" [42]. These differences could trigger difficulties in OMT management and in therapeutic relationships.

In literature, barriers to OMT are explored, but in majority regarding access to care for PWUD, and care provider training, or care organization [45, 46]. There is very little data in the literature on patients’ points of view on their OMT, and on the management of their own treatment in a patient-centred approach. A previous exploratory study was conducted, showing that a majority of PWUD identified some benefits in their OMT [36], as reported in the literature [44], but they were ambivalent regarding this treatment, because they thought it was not like other medical treatments, and 21% of the sample thought it was like taking drugs [36]. PWUD points of view could influence therapeutic relationship, OMT adherence, as OUD is a chronic disorder that requires an active implication of PWUD and a patient centered management.

The aim of our study was to explore the points of view of PWUD treated with OMT for at least six months, in a large multicentre sample.

## Material and methods

### Procedure and ethics

OPAL (NCT01847729) was an observational, cross-sectional, multicentre study involving 10 centres located in the Western region of France. It combined a clinical evaluation [47] and a pre-specified ancillary pharmaco-genetic study [48]. OPAL was conducted in accordance with the Good Clinical Practice Guidelines and the Declaration of Helsinki and was approved by the local ethics committee. Written informed consent was collected from all participants.

### Participants

The study included PWUD 18 years or older, receiving OMT (methadone or buprenorphine) for at least 6 months for opioid dependence (according to the *DSM-IV*) [49]. The 6-month period was chosen since it is the time generally required to adapt and stabilize the dosage of OMT in the context of a global opioid use disorder treatment. In a previous study, we showed that PWUD with opioid dependence were clinically improved and stabilized in the first 6 months and 50% were referred within 280 days of entry into the center specialized in addictive disorders and the OMT introduction [50]. PWUD attending one of the 10 involved centers during the inclusion period, and corresponding to the inclusion criteria were proposed to participate to the study. Exclusion criteria were difficulties reading or writing French and under the guardianship of someone due to diminished capacities.

### Measures

The clinical evaluation consisted of a hetero-assessment (structured interview conducted by one of the investigators) and a self-assessment (self-administered questionnaires completed by the patient). Different clinical data were collected, including impulsivity and ADHD evaluations. The results have been published previously [47]. Only variables that were used for the present analyses are presented in this article:

#### Sociodemographic characteristics

We collected data on gender, age, educational level, marital and parental status, housing, social support, professional status, and financial situation.

#### OUD characteristics

Data concerning the main substance used was collected (heroin, non-medical use of codeine, morphine, buprenorphine, and methadone), the main route of administration (nasal, intravenous, inhaled, oral), and negative consequences related to opioid dependence (financial, social, affective, psychiatric, professional, legal, physical problems).

OMT characteristics were collected (type of medication, duration, OMT initiation with daily supervised dosing by a qualified health professional, current daily dose, compliance, withdrawal symptoms, current opioid use despite OMT or opioid abstinence, defined as the self-reported absence of opioid use over the previous 6 months).

#### Use of other substances and gambling habits

PWUD were asked about the current frequency of any co-addictions (nicotine/alcohol/gambling/illicit substance use). Current co-addictions were considered in case of nicotine dependence (Fagerström nicotine dependence test [51]), and/or high-risk substance use and/or alcohol misuse ( CRAFFT [52]) and/or gambling disorders (The Lie/Bet questionnaire [53]). Detailed method and results for each co-addiction have been published previously [47].

#### Points of view about OMT

A questionnaire was developed by the scientific committee of the study to explore the patients' subjective points of view (Details in “Appendix”). It is a multi-dimensional questionnaire, consisting of 21 statements expressing points of view on OMT, its efficacy, benefits and side effects and its prescription and delivery patterns (see “Appendix”). This questionnaire was developed taking data from the literature into account, the patients’ specific points of view regarding OMT treatment [54] and point of views of PWUD in clinical practice and on PWUD forums (ASUD). It had been tested beforehand in an exploratory study [36].

For each of the items, the PWUD were asked to express their level of agreement: totally agree/agree/disagree/totally disagree.

### Statistical analysis

All statistical analyses were carried out on R statistical software (R Core Team).

A descriptive statistical analysis of the sociodemographic and clinical characteristics was conducted for the entire sample. Continuous variables were described by means and standard deviations if Gaussian and by quantiles otherwise. Categorical variables were presented by numbers and percentages.

An exploratory factor analysis was then performed to explore the structure of the items reflecting the patients’ points of view concerning OMT. A principal component analysis with varimax rotation was performed to explore the structure of variables related to patients’ opinions on OMT. Factor loadings were considered as meaningful if they were above a 0.3 threshold in absolute value. The assumption was that there would be two components, one related to the perception of OMT as a medication as opposed to a drug, and one related to the perception of OMT as a means of social control as opposed to a means of controlling addiction.

## Results

### Descriptive analysis of the sample

#### Sociodemographic characteristics

263 PWUD (opiate-dependent) were included in the study. Descriptive data of the sample is presented in Table 1. Three quarters of the sample were men. The majority had an educational level lower than year 12, and were unemployed. Nearly all participants had stable housing, and one third were in a relationship. Almost all participants reported having social support. However, three-quarters had close friends who use drugs.

**Table 1:** Descriptive data of the sample (n = 263)

	% (n) or Mean ± SD
Sociodemographic characteristics	
Gender (% males)	75 (197)
Age (years)	34.9 (7.4)
Living conditions	
Marital status (% living as a couple)	39% (102)
Stable housing	89% (233)
Social support	92% (240)
Friends who use drugs	95% (181)
Educational attainment (% > 12 years)	13% (34)
Work status and financial situation	
Active workers	46% (121)
On social benefits	36.9% (92)
No income	4.9% (12)
Debt	36% (95)
Co- addictions Nicotine dependence and/or high risk of substance use and/or problem gambling	97% (n = 232)
Opioid use disorder characteristics	
Opioid mainly used	
Heroin	90% (237)
Codeine or morphine	6% (16)
Buprenorphine	3% (8)
Varying	1%(3)
Main route of administration	
Nasal	60% (159)
Intravenous	23% (61)
Inhaled	11% (29)
Oral	4% (10)
Varying	0.4% (1)
OUD damage	
Financial	71% (187)
Social-affective	70% (184)
Psychiatric	69% (182)
Professional	55% (145)
Legal	48% (127)
Somatic	33% (87)
Current OMT	
MTD	68%(178)
BHD	32%(84)
OMT initiation with supervised daily dose	80% (210)
Current daily dose (mg/day)	
MTD	57.4 (32.7)
BHD	7.4 (5.5)
OMT length (months)	51.0 (4.3)
Poor adherence	7% (16)
Current opioid abstinence (excluding OMT)	75% (196)
Successful OMT*	40%(101)
Unsuccessful OMT**	60%(151)

*, ** It corresponds to a definition of what we defined as success or unsucessful treatment

##### OMT, opioid maintenance therapy; OUD: opioid use disorder

Successful or unsuccessful OMT defined in a previous article [47]: *OMT Success defined as an Opioid abstinence without other substance use or gambling**getting worse *Unsuccessful OMT* defined as either *Persistence of opioid use without other substance use or gambling getting worse (11%, n* = *11), or Persistence of opioid use with other substance use or gambling getting worse (10%, n* = *26) or Opioid abstinence but other substance use or gambling getting worse (33%, n* = *84).*

#### Patient viewpoints on their OMT

A descriptive analysis of patient viewpoints on their OMT is presented in Table 2.

**Table 2:** Patient viewpoints on OMT

Items	Strongly agree % (n)	Agree % (n)	Agreement (strongly agree + disagree) %	Disagree % (n)	Strongly disagree % (n)	Disagreement (strongly agree + disagree) %
**OMT efficacy**						
MTD more efficacious than HDB	54 (108)	25.5 (51)	79.5	12.0 (24)	8.5 (17)	20.5
OMT efficacy mainly for withdrawal symptoms	60.4 (142)	31.2 (73)	91.6	5.9 (14)	2.5 (6)	8.4
Global better health since OMT	51.7 (123)	34.8 (83)	86.6	10.5 (25)	2.9 (7)	13.4
OMT has changed lots of things in my life	56.9 (136)	35.9 (86)	92.8	5.8 (14)	1.4 (3)	7.2
OMT has induced health disorders	2.9 (7)	8.5 (20)	11.4	31.7 (75)	56.9 (135)	88.6
OMT has enabled me to stop heroin	63.8 (148)	25.0 (58)	88.8	6.1 (14)	5.1 (12)	11.2
**OMT objectives**						
Objective is to stop heroin	78.8 (186)	13.9 (33)	92.7	2.1 (5)	5.2 (1)	7.3
Objective is to prevent relapse	73.1 (174)	20.6 (49)	93.7	2.1 (5)	4.2 (10)	6.7
Stopping OMT not an objective for me	10.0 (24)	14.2 (34)	24.2	26.4 (63)	49.4 (118)	75.6
**OMT considered as a treatment or a drug**						
OMT is treatment of a chronic disorder	23.2 (54)	39.0 (91)	62.2	18.1 (42)	19.7 (46)	37.8
OMT important in public health to reduce risks for drug PWUD	62.7 (150)	31.4 (75)	94.1	1.7 (4)	4.2 (10)	5.9
OMT is drug replacement	13.6 (32)	27.4 (65)	41.0	38.8 (92)	20.2 (48)	59
Pharmaceutical presentation involves risk of misuse (VN, IV)	17.1 (40)	31.7 (74)	48.8	26.3 (61)	24.9 (58)	51.1
OMT is a drug	16.8 (40)	27.8 (66)	45.6	26.7 (63)	28.7 (68)	55.4
OMT is a treatment like any other	32.5 (78)	25 (60)	57.5	25 (60)	17.5 (42)	42.5
**OMT and relationships with health professionals and relatives**						
Have felt judged by relatives/OMT	27.8 (65)	36.7 (86)	64.5	17.1 (40)	18.4 (43)	35.5
OMT delivery in a pharmacy seen as a legal "deal"	10.2 (24)	16.9 (40)	27.1	32.6 (77)	40.3 (95)	72.9
Have felt judged by a health professional	21.7 (52)	29.7 (71)	51.4	20.5 (49)	28.1 (67)	48.6
Feelings that health professional are not well informed on OMT	28.4 (67)	33.4 (79)	61.8	19.6 (46)	18.6 (44)	38.2
Pharmacist provides important support in follow up	25.4 (60)	35.4 (84)	60.8	20.2 (48)	19.0 (45)	39.3
Physician prescribing OMT provides important support in follow up	63.0 (150)	31.1 (74)	94.1	4.6 (11)	1.3 (3)	5.9

Bold correpond to items category

A Principal Component Analysis led us to prefer a three-component model rather than the expected two-component model. The three components explained 33.62% of the total variance. The factor loading estimates for the three-component solution after varimax rotation are presented in Table 3. The exploratory factor analysis revealed 3 factors:

- The first factor (F1) included items related *to points of view concerning the objectives and efficacy of OMT.*
- The second factor (F2) included items related to the *legitimacy of OMT as a treatment as opposed to a drug.*
- The third factor (F3) included *items related to experiences and relationships with OMT use*.

Finally, four items did not load on any of the three components.

**Table 3:** Factor loadings after varimax rotation

Variables	Loading factors		
	F1	F2	F3
Objective is to stop heroin	**0.667**	0.168	− 0.203
Objective is to prevent relapse	**0.647**	0.159	− 0.28
Efficacy in stopping heroin use	**0.687**	− 0.091	− 0.072
Efficacy on withdrawal symptoms	**0.359**	0.318	0.054
Global positive Impact of OMT	**0.606**	− 0.209	0.239
OMT improves health	**0.645**	− 0.237	0.106
OMT important in a public health perspective	**0.304**	− 0.074	0.072
In follow up, support of physician prescribing OMT is important	**0.33**	− 0.232	− 0.073
OMT is a drug replacement	− 0.08	**0.657**	0.007
OMT is a drug	− 0.134	**0.778**	0.028
OMT delivery in a pharmacy is like a legal deal	− 0.04	**0.657**	0.121
OMT is a treatment like any other	0.032	− **0.473**	− 0.089
OMT in capsules or tablets could promote snorting or IV use	− 0.193	0.122	**0.311**
Has felt judged by relatives	− 0.003	− 0.021	**0.651**
Has felt judged by health professionals	0.06	0.055	**0.766**
Feeling that health professionals are not well informed on OMT	0.064	− 0.027	**0.666**
OMT is treatment of a chronic disorder	0.105	− 0.218	0.199
Stopping OMT is not an objective for me	− 0.171	− 0.195	− 0.234
Pharmacist’s support is important in follow up	0.078	− 0.09	− 0.158
OMT induces health disorders	− 0.218	0.179	0.229

## Discussion

The main objective of this study was to collect points of view on their treatment from PWUD on OMT prescribed for an OUD. Points of view were collected from 263 opioid-dependent patients.

### A globally positive perception of OMT and medical support

The main result of the study was that most PWUD had positive points of view on the efficacy of their OMT. First, a great majority identified a positive impact on their lives, as 92.8% agreed or strongly agreed that OMT had changed a lot of things in their lives. Only 11.2% agreed or strongly agreed that OMT had induced health disorders. OMT occasionally causes severe side effects, (sweating, libido disorders, weight increase…) that can deteriorate patients’ quality of life [42], which may have led some of our PWUD to drop out [55]. However, it could be suggested that they did not identify these side effects as health disorders, or another hypothesis is that in our sample, where the average duration of OMT intake was 4 years, side effects could have influenced early drop-out from follow-up by some OMT PWUD. The perceived benefits by PWUD can be set against the positive impact of OMT on quality of life reported in the literature [56, 57], more particularly when combined with psychosocial interventions [58–61].

Indeed, OMT is a pharmacological treatment, but it is not just that [62]. Most of the time, dependent opioid-dependent PWUD have a combination of significant mental health, physical health and social adjustment problems that can badly influence their prognosis and interfere with their recovery [63]. Specialised centres, support and treatment programs are necessary for the most severe opioid-dependent PWUD to retrieve freedom from the compulsion to use opioids, like heroin [62]. Our sample comprised 90% heroin PWUD (snorting or intravenous for the majority), alongside a high level of addictive comorbidities for 97% of the sample, a high prevalence of lifetime ADHD (44%) [47], and social vulnerabilities, including a low educational level, social isolation, and unemployment, making them a group at higher risk. The severe medical profile of the sample was not surprising, as the majority (68%) of the PWUD included in the sample were prescribed methadone, which is indicated for the most severe PWUD [33] and the recruitment was carried out in specialized centres (addictive disorder centres or hospitals). Psychosocial measures, which are well developed in these highly structured and regulated programs, were confirmed by 80% of our sample reporting a daily issue of their treatment at initiation. Supervised daily issue is more suitable for the at-risk PWUD and is not systematically applied in case of pharmacy-based delivery of BHD [62]. Concerning the patients’ point of view on psychosocial support, medical support was considered important for 94.4% of the sample, who agreed or strongly agreed that the physician prescribing their treatment provided important support for them. This finding could reflect a good relationship between physicians and patients, which is consistent with the treatment model defined by Dole and Nyswander. According to them, the main objective of MTD treatment is the management of a chronic disease by medication, and one main element is the therapeutic relationship [64]. Pharmacists could also be an important source of support, especially in France, where they are responsible for BHD delivery, and also MTD dispensed in pharmacies. In our sample, a majority agreed but 39.2% disagreed or strongly disagreed that the pharmacist’s support was important in their OMT follow-up. The main hypothesis is that because the medical profiles of the sample were severe, and the PWUD were recruited in specialized centres with supervised OMT delivery, pharmacists were not involved in the management of OMT. The second hypothesis is that the pharmacists were insufficiently implicated, or identified by physicians and by health professionals, as being potentially helpful in the management of OMT.

### OMT and relationships with health professionals and relatives

Regarding relationships with health professionals, we also showed that 51.4% of the sample agreed or strongly agreed that they had already felt judged by health professionals for their OMT. In addition, 61.8% of the sample agreed with the feeling that health professionals did not seem sufficiently informed on OMT. These results could seem surprising, as OMT has now shown considerable benefit to public health, and has been authorized in France for more than 20 years. But it could also reflect the fact that addiction is still not considered as a chronic disease, and opiate use disorders are seen as a social problem rather than a medical problem [65].

It could also reflect a lack of training of health professionals on this topic. Many physicians feel ill-prepared when prescribing OMT [66] or lack sufficient training and experience. Only a minority of early career family physicians report receiving adequate preparation to prescribe buprenorphine treatment in the course of their training [30].

In the PCA, the items concerning feelings of being judged by health professionals, and feelings that health professionals were not sufficiently informed about OMT, were correlated with the item about feelings of being judged by relatives. These items constitute a "relationship factor" in our study, and could reflect the patients’ personal experiences of their treatment, and the fear of being judged. The question of identity is central to the management of OUD. Relating to an "addict identity" is exciting, it is associated with a free spirit and is not bound by conventional expectations, which contrasts with PWUD who relate to a treatment identity. OMT can be perceived as a loss of autonomy [67] by the PWUD themselves and drug consumers around them, and in our sample 95% of PWUD said they had friends who use drugs. In contrast, OMT could also be associated with a negative image of drug dependence for those who wish to escape from this image [36, 54]. Subjects undergoing OMT feel that this treatment keeps them dependent, and wish to continue the treatment in the short or medium term only so as to subsequently achieve total abstinence from all opioids [68]. In our sample, only 24.2% agreed or strongly agreed with the idea that stopping OMT was not an objective for them, like the preliminary study where a large minority of the sample did not think about stopping the treatment [36].

Regarding pharmaceutical diversion of OMT, it is considered no more considered in literature as a "problem" but as an alternative perspective in the PWUD trajectories [54, 69]. Diversion of OMT is an inevitable question in OMT management, and effective measures include supervised OMT issues, restrictions on unsupervised OMT if PWUD do not present stability criteria, and awareness about the galenic characteristic of take-home OMT [62]. We may suppose that because being aware of the risks of diversion according to the galenic presentation of OMT was also associated with the "relationship factor" in the factorial analysis, PWUD who felt judged were particularly aware of the medical concern of substance diversion. The perceived risks of diversion have an influence on medical decisions and modes of delivery, and this could be perceived by PWUD as a judgement or as a lack of confidence in their ability to change [70].These results correspond to the need to consider differently the pharmaceutical diversion, as identified by Bardwell et al. [69]. They underlined the need to develop strategies that engender greater safety, reduce drug harm, and alleviate the effects of these constraints, including through policies promoting safer drug supplies, decriminalization, and employment [69].

### OMT: a treatment for a chronic disorder

The views and medical training on OUD treatments need to change, and more particularly concerning heroin dependence [62]. OUD treatment is envisaged in emergency contexts, when withdrawal symptoms are painful and difficult to manage [71], and when the risk of death from overdose are high, with either illicit or prescribed opioids [72, 73]. OMT treatments have been authorized in many countries, as in France, in the context of a public health crisis with virus transmissions and an epidemic of overdoses [29]. But the treatment of OUD is better conceptualized as the management of a chronic condition [74]. In our study, the patients’ points of view on the question of OUD and OMT chronicity were not homogeneous, a majority agreed or strongly agreed that OMT was a treatment for a chronic disorder, but 37.8% disagreed with this statement. However, concerning patient expectations and points of view on OMT efficacy, the results are coherent with this chronicity paradigm. First, patients’ expectations concerned the objective of discontinuing heroin for 92.7% of the sample, and preventing relapse for 83.7%. These items were statistically correlated with an "efficacy factor" of OMT in the factorial analysis. This factor includes these 2 objectives and the efficacy of OMT on withdrawal symptoms and on stopping heroin consumption, the positive impact on their life and on their health and also the global perception of OMT as an important public health issue for risk reduction among drug PWUD. Long term benefits were identified by patients in our study, but OMT is often perceived as preventing negative outcomes, it is not necessarily seen as producing positive outcomes for the individual, such as better health or quality of life [75]. In prison, benefits of OMT are largely framed in terms of avoiding negative experiences or outcomes, rather than as direct positive benefits of treatment. These perceptions correspond to the considerable ambivalence that many opioid dependent persons have towards OMT [76]. This difference in the benefits perceptions may be explained by the median duration of OMT in our study, which was 4 years.

We also showed the importance of support from physicians prescribing OMT, which was associated with the "efficacy factor". This identified factor showed that PWUD had integrated the short-term objective of alleviation of withdrawal symptoms, which is an important step in treatment, as failure in this management is associated with poorer outcomes [77]. But PWUD also integrated a combination of long-term objectives sought through OMT [62], including risk reduction, improvement in mental health and medical situations, and the restoration of their of impaired social role, with the psychosocial support of the physicians prescribing their OMT, appearing as a central variable in OMT efficacy.

### Is OMT a treatment or a drug ?

In our study, as shown in the preliminary study [36], the patients’ points of view on OMT as a drug or a treatment like any other were ambivalent. In the descriptive analysis, 57.5% agreed that OMTs were treatments like any others, but 40.9% agreed or strongly agreed that OMTs were drug replacements, 44.6% agreed that OMTs were drugs, while only 27% agreed that OMT delivery at a pharmacy felt like a "legal deal". In the factorial analysis, these three items were combined in a third " treatment factor". It also included at the extreme opposite of this "treatment factor" the perception of OMT as a treatment like any other.

This "treatment factor" and the patients’ points of view correspond to an axis previously defined in the literature concerning the role of OMT perceived by PWUD and also by physicians either as a medical treatment or as a legal drug [30, 42, 54]. The changes expected after treatment initiation for an OUD are changes in attitudes, behaviour and identity "from drug addict to patient" [64]. In the course of these changes, OMT can be perceived and consumed by PWUD as a drug or rather as a treatment, and this can change depending on the individuals and according to the time in each individual’s life [54]. In therapeutic relationships, it is important to consider and accept patients’ intermediate objectives. Their motivation is central to treatment compliance, and there are discrepancies between the medical perception of OMT benefits and success and patient perceptions. In our study, only 40% responded to the medical criteria for successful OMT, but a majority of PWUD identified great benefits in their OMT. Trujols et al*.* showed that in a methadone maintenance treatment program, there was low concordance between the patients’ perceptions and the clinical staff’s perceptions of improvement [34]. One hypothesis concerning these results is that OMT objectives are not sufficiently negotiated and agreed on with PWUD, implicitly and naively assuming that PWUD and clinicians share the same objectives [34, 78]. In addition, despite a model that provided for broad availability of OMTs [31], MTD and BHD are authorized for maintenance, but no other pharmacological alternatives, such as diamorphine or slow-release oral morphine, are authorized in France. These pharmacological tools, when monitored and supervised, have proved useful as an incentive for treatment initiation and for the development of patient implication and autonomy in their treatment decisions [62].

Another variable that could influence patients’ perceptions of OMT is the way OMT is prescribed. Despite the public health impact, patterns of OMT prescription can lead to poor quality treatment. Inadequate doses of OMT can contribute to poorer outcomes, and to treatment dissatisfaction among PWUD [79]. Guidelines on OMT recommend MTD doses in the 60–100 mg per day range and BHD doses from 8 to 12 mg per day [25].These doses should be increased in case of severe OUD and dual disorders[80], but in our medically severe sample where addictive comorbidities were highly prevalent, the average daily dose of OMT was below the recommendations. This sub-therapeutic dosage in heroin-dependent PWUD has already been reported in the literature, as in England where in one study the mean methadone dose was 56 mg per day [81] and in another more than 60% of PWUD in treatment reported using heroin in the previous month [82]. In our study, it could be suggested that PWUD were stabilized: 75% were not currently consuming heroin, and had been in treatment for 4 years, with good compliance, which could explain OMT dose reductions, despite a high level of addictive comorbidities (97%).

Another hypothesis is that this situation is the result of differences between 2 theoretical points of view on OMT. Dole and Nyswander suggested a "medical model" of OUD treatment, in which methadone was a medication for treating an acquired disease [62, 64]. In this pharmacological theory of OMT as treatment for a brain disease, one of the key issues is that physicians and PWUD have to understand and accept that OMT is primarily a pharmacological treatment. But another theory still remains, favouring the psychotherapeutic paradigm, where OMT prescription is considered as an opportunity to maintain contact with the PWUD and the delivery program as a way of helping psychosocial rehabilitation [62, 83]. In this paradigm, part of the centres’ role is to discipline deviant individuals, and offering alternative medications can be seen as over-indulgent [77].

### Strengths and weaknesses

This study collected a large dataset concerning PWUD with OUD and OMT. To the best of our knowledge, this is the first study to explore this topic in such a broad manner, as this topic is rare in the literature, particularly with such a large sample. The PWUD included filled in the self-report questionnaire on their points of view on OMT, which could reflect patients’ interest in this topic. Despite a single-nationality sample, which could limit the representativeness and extrapolation of the results, our recruitment was multicentre with a variety of centres with different practices (hospital units, health centres, prisons, etc.). This provided heterogeneity in the population. Heterogeneity of the sample was intended in order to improve its representativeness of real-life OUD PWUD in treatment in specialized centres. Missing data was marginal, and the number of PWUD enabled statistical analyses with sufficient power. This study was based on a global evaluation of the PWUD and an exhaustive assessment of substance and behavioural addictive disorders.

Our study has certain limitations. First, its design was cross-sectional with recruitment in specialized centres. The sample combined many severity factors, and was not representative of the PWUD under OMT in France, and the sample did not match national data, where the majority of opiate-dependent PWUD receive buprenorphine via pharmacy-based delivery, prescribed for two-thirds by family physicians [30, 84, 85]. In addition, the multiplicity of recruiting centres and practitioners meant that we could not provide participation or refusal rates. Second, we deliberately built a semi-structured interview to explore OUD characteristics (and especially its negative consequences, psychiatric repercussions for example). We made this decision because no validated scale existed. Moreover, the criteria of time could influence results, and the findings might differ among patients on OMT over one year of treatment.

## Conclusion

The results of this study underline how patient expectations and points of view on efficacy are coherent with and correlated to the pharmacological benefits of OMT, and to the combined psychosocial measures. Patient implication in treatment decisions, a patient-centred approach to individualized goals, with an adaptation of pharmacological prescription and delivery modes could certainly help enhance positive points of views among PWUD on OMT and help PWUD towards their recovery.

## Data Availability

All datasets generated for this study are included in the manuscript.

## Appendix: Items of the questionnaire

*5 items concerned points of view on OMT efficacy*: MTD more efficacious than HDB/efficacy on withdrawal symptoms/efficacy on discontinuing heroin consumption/global positive impact of OMT/OMT and improved health/OMT-induced health problems.

*3 items concerned points of view on the objectives of OMT*: the objective is to quit heroin/the objective is to prevent relapse (heroin)/discontinuing OMT is not an objective for me.

*6 items concerned points of view on OMT as a treatment or a drug*: OMT is a treatment for chronic OUD/OMT treatment is important in a public health perspective to reduce risks for drug PWUD/OMT is a replacement drug/OMT is a drug/OMT is a treatment like any other/the presentation of OMT in the form of a capsule or a tablet can promote snorting or intravenous use.

*6 items concerned relationships with relatives and health professionals*: has already felt judged by relatives for their OMT/OMT delivery in a pharmacy is like a legal "deal"/Has already felt judged by a healthcare professional for their OMT/Feelings that some healthcare professionals are not well informed about OMT/During follow-up care, pharmacists’ support is important/During follow-up support from the physician prescribing OMT is important.

## Abbreviations

OUD: Opioid Use Disorder
OMT: Opioid Medication Therapy
MTD: Methadone
BHD: Buprenorphine
